# Recent Progress on Bioresorbable Passive Electronic Devices and Systems

**DOI:** 10.3390/mi12060600

**Published:** 2021-05-22

**Authors:** Zhihuan Wei, Zhongying Xue, Qinglei Guo

**Affiliations:** 1 School of Microelectronics, Shandong University, Jinan 250100, China; 201912227@mail.sdu.edu.cn; 2 State Key Laboratory of Functional Materials for Informatics, Shanghai Institute of Microsystem and Information Technology, Chinese Academy of Sciences, Shanghai 200050, China; 3 State Key Laboratory of ASIC and Systems, Fudan University, Shanghai 200433, China

**Keywords:** bioresorbable electronics, passive electronic components, bioresorbable materials, device architectures, biomedical applications

## Abstract

Bioresorbable electronic devices and/or systems are of great appeal in the field of biomedical engineering due to their unique characteristics that can be dissolved and resorbed after a predefined period, thus eliminating the costs and risks associated with the secondary surgery for retrieval. Among them, passive electronic components or systems are attractive for the clear structure design, simple fabrication process, and ease of data extraction. This work reviews the recent progress on bioresorbable passive electronic devices and systems, with an emphasis on their applications in biomedical engineering. Materials strategies, device architectures, integration approaches, and applications of bioresorbable passive devices are discussed. Furthermore, this work also overviews wireless passive systems fabricated with the combination of various passive components for vital sign monitoring, drug delivering, and nerve regeneration. Finally, we conclude with some perspectives on future fundamental studies, application opportunities, and remaining challenges of bioresorbable passive electronics.

## 1. Introduction

Transient electronics form the basis for classes of bioresorbable electronic devices or systems that undergo complete physical disintegration and chemical dissolution after a predefined operation period. Compared to permanent electronics, one of the most significant advantages of transient electronics, aiming for biomedical applications, manifests in the elimination of risks and costs associated with the secondary surgery for the device retrieval, thus attracting numerous attentions from both academic and industrial researchers [1,2,3]. As a result, various bioresorbable electronic devices or systems with different diagnosis and/or treatment functionalities are developed, such as intracranial pressure/temperature sensors [4,5], electronic stimulators for nerve regeneration [4,6,7], drug delivery systems [7,8,9,10], and others.

From the viewpoint of device categories, bioresorbable passive electronic components (resistor, capacitor, and inductor), capable of monitoring and/or treating human diseases, are of great interest due to their easy fabrication process, simple device architecture, and legible working mechanism for accurate data extraction. Resistors are commonly used to control the current flow in electronic circuits. Changes in resistivity, length, or cross-sectional area of the material that utilized to construct resistors will induce changes in the resistance, thereby forming the fundamentals of most resistor-type sensors, for example temperature sensors [11] and pressure/stress sensors [12,13,14,15,16]. Capacitors are mainly used for signal filtering or temporary charges storing in electronic circuit. Relative dielectric, area of the parallel electrode pair, and distance between the electrodes strongly determine the capacitance. Changes in the relative dielectric or the distance between two electrodes are widely adopted to construct a capacitor-types sensor for the detection of regional body temperature [17], pressure [18], or humidity [19]. Inductors are able to store the magnetic energy induced by the electric current passing through them. They can be utilized as filters [20] or as antennas for energy harvesting and data transmitting [21]. Moreover, the integration of different bioresorbable passive devices, such as resistor (R), capacitor (C), and inductor (L), could generate a battery-free fully passive system, which enables the wireless power delivery or the wireless data communication through the near-field inductive coupling.

**Figure 1:**
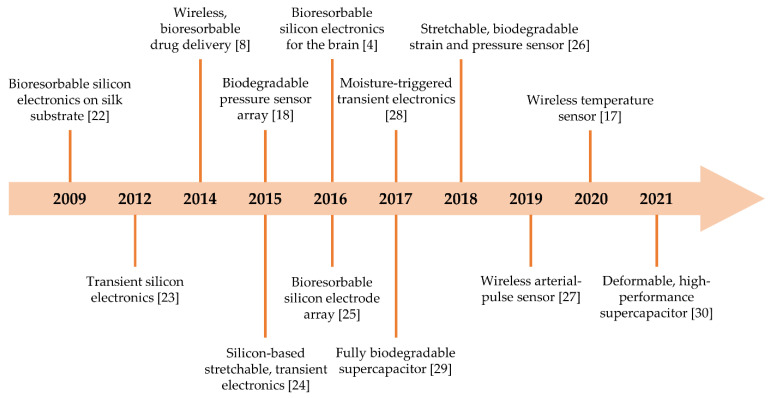
Milestones on bioresorbable passive components or their integrations and the hybrid with actively electronic devices.

Milestones of these passively electronic components or their integrations and the hybrid with actively electronic devices appear in Figure 1. The first demonstration of bioresorbable silicon electronics was realized by Kim et al. [22], in which silicon-based active electronics integrated with passive devices was fabricated on a silk substrate. Then, a fully transient form of silicon electronic system consisting of various active and passive devices was proposed by the Prof. J. A. Rogers [23], and all involved materials were biocompatible and biodegradable. After that, various efforts have been devoted to develop passively electronic devices or systems with different functionalities that can be partly or completely disappear through physical disintegration or chemical reaction after a predefined operation period, such as wireless bioresorbable drug delivery [8], bioresorbable (or wireless) sensors [4,17,18,24,25,26,27], triggerable transient electronics [28], and power supply devices [29,30]. These achievements significantly promote the rapid development of bioresorbable passive electronics, especially for applications in biomedical engineering.

In this review, we will review the recent progress on bioresorbable passive devices or systems, with an emphasis on their applications in biomedical engineering. In the first section, we will summarize the material strategies for the construction of bioresorbable passive devices. Then, examples based on bioresorbable resistors, capacitors, and inductors, such as biodegradable pressure sensors, temperature sensors, pH sensors, bioelectrodes, power supply systems, filters, and antennas, will be successively discussed. After that, battery-free, fully passive and wireless systems fabricated by the integration of bioresorbable passive electronic components (also named as LCR circuits), capable of monitoring vital signs, delivering drugs, and stimulating nerve for regeneration, are discussed. Finally, we conclude with some perspectives on future fundamental studies, application opportunities and remain challenges of bioresorbable passive electronics.

## 2. Material Strategies for Bioresorbable Passive Devices

To fabricate a bioresorbable passive device, both organic and inorganic materials can serve as either functional layers or dielectrics. However, the utilized materials, as well as the chemical products generated from their reactions with the surrounding environment, should be biodegradable, biocompatible, and bioresorbable. Table 1 summarizes the commonly used materials for the construction of bioresorbable passively electronic devices, as well as their key applications, dissolution rate and dissolution condition. Appropriate semiconductors include silicon (Si) [23,31,32,33,34,35,36], germanium (Ge) [32], SiGe alloy [32], zinc oxide (ZnO) [37,38,39,40], and other related inorganic materials, as well as organic semiconductors, such as diphenylalanine polyfluorene polymer (FF:PF) [41], and riboflavin tetrabutyrate (RFLT) [42]. On the basis of their intrinsic physical properties, for example thermosensitive effect and piezoresistive/piezoelectric effect, resistor-based bioresorbable sensors are available for the in vivo monitor of temperature and pressure. In terms of device performances, advantages for inorganic semiconductors mainly lie in their stable and outstanding physical/chemical properties, thus enabling high-performance bioresorbable passive devices. For organic semiconductors, however, their natural flexibility will be one of the most significant advantage especially for fabricating flexible bioresorbable electronic implants, which can compromise the mechanical stress induced by the wriggle of bio-tissues. Besides, the ease of fabrication process, mostly involving printing or coating techniques, also attracts researchers to develop organic semiconductor-based bioresorbable passives devices for biomedical applications.

Another important material for bioresorbable passive devices is the conductor, which can serve as electrodes for both resistors and capacitors, coils for inductors, and electrical interconnections for their integrations. Appropriate metallic materials for bioresorbable conductors include magnesium (Mg) [46,47], zinc (Zn) [14,48,49,50], tungsten (W) [29,40,51,52], iron (Fe) [29,53], molybdenum (Mo) [4,12,15], and their alloys [43,54]. In addition, bioresorbable composite conductors consisting of metallic nano/micro-materials (such as particles and wires) and polymer matrix are also of increasing interest. Similar to organic materials, bioresorbable composite conductors can be produced by convenient printing or coating techniques, thus eliminating the requirement on complex and costly vacuum deposition instruments for growing traditional metal layers. Nevertheless, high-conductivity, low-temperature processing, and long-term stability in electrical properties are required for bioresorbable composite conductors. Various sintering methods, such as electrochemical sintering [55], evaporation–condensation-mediated laser sintering [14], anhydride-assisted spontaneous sintering [56], and photonic sintering [57], have been developed to reduce the processing temperature or to enhance the conductivity. In some specific cases, flexible and stretchable bioresorbable conductors are also demonstrated by pure organic materials [58,59,60,61].

As for bioresorbable dielectrics, the suitable material strategy is primarily determined by the purpose of applications. For example, inorganic materials like silicon oxides (SiO_2_) and silicon nitrides (Si_3_N_4_) can serve as the ideal isolating layer or passivation coating of bioresorbable electronics, though high-temperature deposition techniques, which may not suit for polymer-based substrates, or layer transfer techniques, are involved [34,44,62]. In particular, SiO_2_, Si_3_N_4_, or their hybrid stacks are able to block the penetration of water or ions, which is crucial for the long-term operation of bioresorbable electronics [63]. Biodegradable organic dielectrics, for example, polylactide acid (PLA) [64,65], poly(lactic acid) (PLLA), poly(lactide-*co*-glycolide) (PLGA) [45,66,67], poly(vinyl alcohol) (PVA) [68,69], poly(octanediol-*co*-citrate) (POC) [24], poly(glycerol sebacate) (PGS) [70], polycaprolactone (PCL) [64,71], poly(hydroxybutyrate) (PHB) [72], poly(hydroxyvalerate) (PHV) [18], poly(octamethylene maleate (anhydride) citrate) (POMaC), spin-on glass (SoG) [73], silk [20,22,46,74], and candelilla wax [75], are mostly employed as the substrate or the encapsulation layer of bioresorbable electronics. Notably, the convenience of controlling over the thickness of organic dielectrics, mostly enabled by tuning key parameters during the spin-coating or drop-casting process, allows for adjustable dissolution timescale and dissolution rate of bioresorbable electronics. Moreover, on-demand transient bioresorbable electronics are also available by embedding some specific materials into the organic encapsulations, which is triggerable to destroy the device by external signals [28,76,77,78,79,80,81,82].

## 3. Bioresorbable Resistors

Characterizations on the electrical resistance of materials, with a significant change with external signals, form the basis of sensors. In this regard, the basic electrical component is resistor. By utilizing appropriate materials, bioresorbable resistor-based sensors, capable of monitoring biophysical signals, biochemical signals, and biopotential signals, are fabricated. In the following section, typical examples of these functional bioresorbable resistors will be described.

### 3.1. Pressure/Temperature Sensor

Bulleted Measurements of the pressure in cranium, oculi, blood vessels, thoracic cavity, or bladder are essential for diagnosing diseases, such as traumatic brain injury, brain tumors, hydrocephalus, stroke, glaucoma, hypertension, pneumothorax, and urinary incontinence. Excessive pressures inside the human body may cause potential diseases or physical injuries. Moreover, the flowing observation via pressure sensors in recovering stage after injuries or surgical procedures is also important [83,84,85]. Therefore, continuous efforts have been devoted by researchers to develop bioresorbable pressure sensor for monitoring the pressure inside the human body precisely, continuously, and chronically. In addition, measurements of the internal body temperature also play vital roles in the diagnosis of immune response, monitoring of wound healing, and precise management of disease therapies.

In 2016, Kang et al. reported bioresorbable silicon electronic sensors for continuous monitoring of intracranial pressure and temperature, which is essential for the treatment of brain injury [4]. Fabrication of the sensors starts with placing a silicon nanomembrane (SiNM) based strain gauge on one edge of an air cavity, which is formed by sealing a PLGA film against a square relief-contained (with a depth of 30–40 µm) substrate. The sealed air cavity will induce the deformation of PLGA film when the intracranial pressure changes, thus leading to variations in the conductivity of attached SiNM-based strain gauge. The measured pressure responses are quantitatively consistent with those of clinical-standard intracranial pressure sensors. However, intracranial temperature will also influence the conductivity of SiNM-based strain gauges due to intrinsic thermal sensitive effect of Si. Although high-concentration doping could weaken this thermal sensitive effect, the authors still design a temperature sensor, which is not affected by the pressure, as a compensation, to calibrate the temperature-dependent piezoresistivity. Notably, all utilized materials, such as SiNM, PLGA, nanoporous silicon or Mg substrate, and Mg electrodes, are biodegradable and bioresorbable. Therefore, suitable encapsulation materials that reduces the dissolution of bioresorbable silicon-based sensors are required to provide a long-term stable operation in brain.

**Figure 2:**
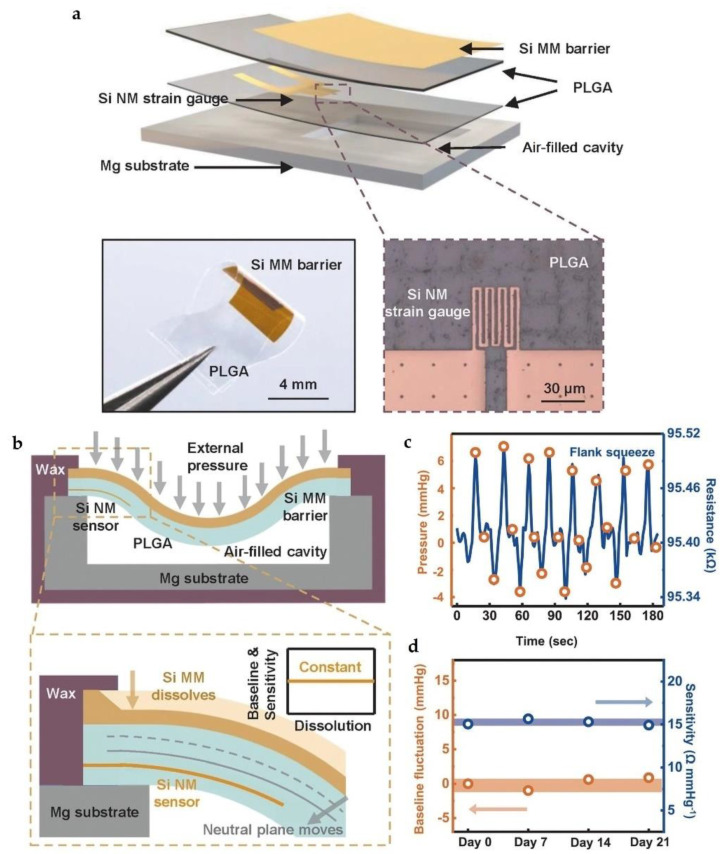
(**a**) Schematic illustration of the pressure sensor with a monocrystalline silicon membrane encapsulation; (**b**) schematic illustration of the cross section. During dissolution process of Si membrane, the neutral plane of the device underwent a displacement; (**c**) in vivo intracranial pressure recording when compressing and releasing the flank of the rats. The data was collected by both biodegradable sensor and commercial sensor; (**d**) the baseline (orange) and sensitivity (blue) remained largely unchanged through flank squeezing process on day 0, 7, 14, 21 after surgery. Reproduced with permission from [86].

In 2020, Yang et al. utilized SiNMs to encapsulate resistor-based bioresorbable pressure sensors, and prolonged stable operational lifetime (up to 3 weeks) was realized, suggesting significant potentials to meet the requirement for clinical applications [86]. The whole bioresorbable system involve material strategies, mechanical design, and multiple sensors. As shown in Figure 2a, the authors used a monocrystalline silicon membrane (~1.5 µm in thickness) as encapsulation layers. Monocrystalline silicon membrane has excellent biofluids proofing performance as well as considerable dissolution rate in biofluids. Blends of natural wax (~300 µm in thickness), consisting of candelilla wax and beeswax, with a weight ratio of 3:2, served as edge-sealing barriers and interlayer bonding to prevent biofluids permeation from periphery and film interfaces, as shown in Figure 2b. The authors also pointed out that the reduction in thickness of SiNM encapsulations during their dissolution in biofluids will push the neutral mechanical plane toward the SiNM strain gauge, thus affecting its responsivity and causing drifts in the operational characteristics. Therefore, optimizations in the mechanical designs are required to restrain drifts in sensor performance during the dissolution process. Guided by theoretical calculations and finite element analyses (FEA), the authors demonstrated that if the strain sensor was placed at the one-third position of the PLGA layer, the sensitivity would remain unchanged during the entire dissolution process. In vivo recordings of the intracranial pressure in an animal model were performed by utilizing the developed bioresorbable sensors, as shown in Figure 2c, which agrees well with the results obtained from the commercial sensor. Moreover, by utilizing SiNM as the encapsulation layer, the bioresorbable sensor could stably operate over 3 weeks, as demonstrated in Figure 2d.

For scenarios like nursing of traumatic brain injury (up to one week) and glaucoma (several months), long-term stable operation of bioresorbable sensors, with strong biofluid barriers that prevent unwanted degradation of the active elements are required [87]. In 2018, Shin et al. demonstrated resistor-based bioresorbable pressure sensors packaged with thermally grown silicon dioxide (t-SiO_2_, thickness ~ 10 nm) [5]. It turned out that t-SiO_2_, with few defects, exhibited an extremely slow degradation rate, thus enabling excellent perfection over large areas. The whole resistor-based bioresorbable system includes two pressure sensors and two temperature sensors. The working principle for sensing the intracranial pressure is similar to the work reported by Kang et al. [4] that two pressure sensors are placed on the top surface of an air cavity, and other two temperature sensors locate on the substrate that close to the air cavity, so the coupling effect of pressure on the temperature sensor can be eliminated. The in vivo experiments of intracranial pressure monitoring in rats suggest that the device could work with a high level of accuracy and extremely low baseline drifts for about 25 days. The whole device would be fully dissolved at around 400 days in artificial cerebrospinal fluid at 37 °C, and the dissolution time can be reduced to about 290 days without significantly compromising the sensor response by optimized designs of device architectures.

### 3.2. PH Sensor

**Figure 3:**
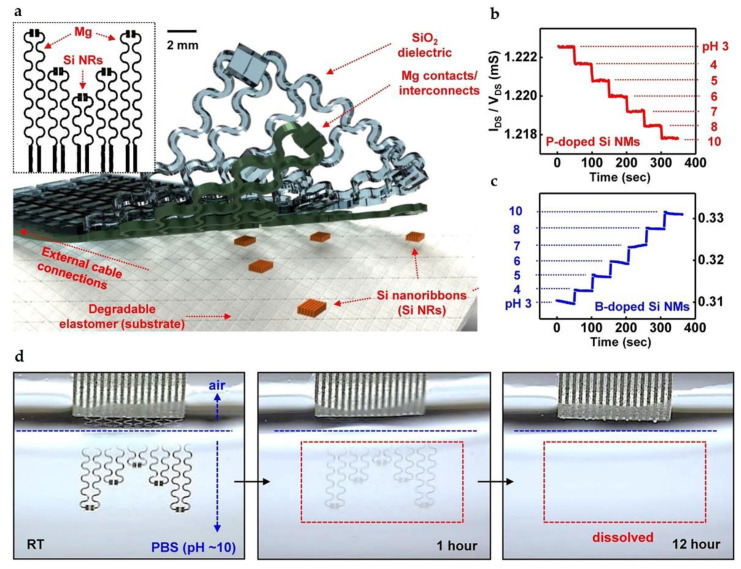
(**a**) Schematic illustration of the biodegradable pH sensor; (**b**) illustration of changes in conductance of phosphorus doped Si NRs in different pH solutions (from 3 to 10); (**c**) illustration of changes in conductance of boron doped Si NRs in different pH solutions (from 3 to 10); (**d**) images of the dissolution process with the device immersing in PBS (pH = 10) at room temperature. Reproduced with permission from [24].

Variations in the pH value of biofluids are useful for diagnosing the human health status [88]. For example, the pH value of sweats has a strong relationship with the dehydrated condition or the perspiration rate [89]. In 2015, Hwang et al. reported a resistor-based stretchable and biodegradable pH sensor consisting of functionalized silicon nanoribbons (SiNRs), Mg contacts, and SiO_2_ interlayer dielectrics and encapsulations, as schematically illustrated in Figure 3a [24]. Both phosphorus- and boron-doped SiNRs (with a doping concentration of about 10^20^/cm^3^) undergo conductance changes when exposed to solutions with different pH values. Here, the functional groups on the surface of SiNRs are protonated at low pH or deprotonated at high pH, thus leading to the depletion or the accumulation of charge carriers, respectively. In aqueous solutions with pH from 3 to 10, the conductance of phosphorus-doped SiNRs decreases with a sensitivity of 0.1 ± 0.01 μS/pH (Figure 3b), while the conductance of boron-doped SiNRs gradually increases with a sensitivity of 0.3 ± 0.02 μS/pH (Figure 3c). The authors also demonstrated that bioresorbable pH sensors produced with heavily boron-doped SiNRs could work stably (undergo 1% or fewer conductance change) in phosphate buffer saline (PBS) with a pH value of 7.4 and a temperature of 37 °C for five days. The transience behavior of resistor-based biodegradable pH sensors was investigated by immersing them in PBS (pH = 10) at room temperature, as illustrated in Figure 3d, which completely disappear after a 12 h immersion. Similar bioresorbable pH sensor was also demonstrated by Kang et al. [4] with phosphorus-doped SiNRs, which were functionalized with 3-aminopropyltriethoxysilane. In terms of practical applications, current resistor-based bioresorbable pH sensors require encapsulations to prolong the time duration for stable operation. However, systematic studies about the influence of encapsulations on the performance of pH sensors, such as sensitivity and response time, remain rare.

### 3.3. Bioelectrode

Bioelectrodes are conductors that serve as the interface between biological organs/tissues and external electrical systems. The primary function of bioelectrodes lies in sensing or measuring electrophysiological activities of biological organs/tissues, therefore, electromyogram (EMG), electrocardiogram (ECG), electroencephalogram (EEG), electrooculogram (EOG), and other related, can be extracted for diagnostic purposes [90,91]. Another important function of bioelectrodes involves the introduction of electrical stimulations into biological organs/tissues for therapeutic purposes, especially for treating neurological disorders, such as epilepsy, Parkinson’s disease, and depression [92,93,94,95].

**Figure 4:**
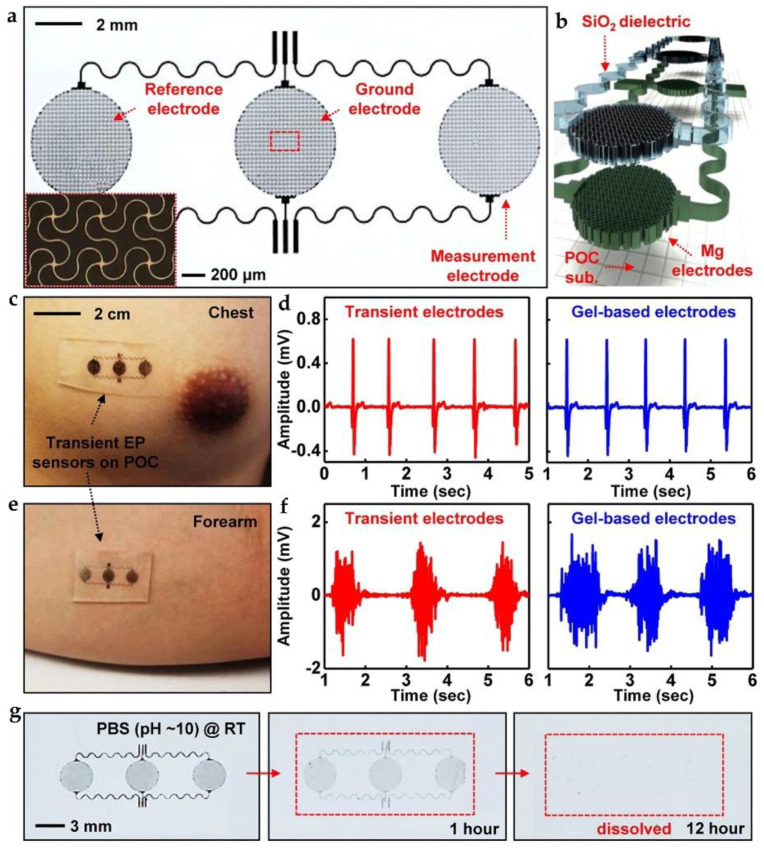
(**a**) Optical image of the biodegradable EP sensor; (**b**) exploded view of the device; (**c**) photograph of a device attached to the chest to measure ECG; (**d**) ECG collected by a biodegradable sensor and a standard gel-based device; (**e**) photograph of EP sensor located in forearm to measure EMG; (**f**) EMG collected by a biodegradable sensor and a standard gel-based device; (**g**) dissolution process of the EP sensor immersed in PBS (pH = 10) at room temperature. Reproduced with permission from [24].

In 2015, Hwang et al. reported a stretchable, biodegradable electrophysiology (EP) sensor [24]. As shown in Figure 4a, the EP sensor consists of filamentary Mg serpentines (thickness = 300 nm), SiO_2_ (100 nm in thickness), and a POC substrate. Mg serpentines serving as the bioelectrodes were mounted on the skin through the POC substrate, and the measured electrical biopotentials are mainly contributed by the displacement currents, which are generated by capacitive coupling through the SiO_2_, as shown in Figure 4b. High-quality recordings of ECG (left of Figure 4d) and EMG (left of Figure 4f), comparable to those recorded by conventional gel-based electrodes (right of Figure 4d,f), are realized by the fabricated biodegradable EP sensors on the chest (Figure 4c) and the right forearm (Figure 4e), respectively. Figure 4g shows the transience process of the EP sensor immersed in PBS (pH = 10) at room temperature. Mg bioelectrodes will be firstly dissolved within hours, then complete dissolution occurs after days/weeks due to relatively slow dissolution rate of SiO_2_. In this case, the developed biodegradable EP sensor is utilized as disposable wearable electronics rather than long-term monitoring.

For some other cases that involve chronic diagnosis and treatment, such as monitoring postoperative seizures or brain activities after epilepsy surgery, bioresorbable bioelectrodes need to work stably for several weeks or even several months [96]. Under these circumstances, traditional bioresorbable metals, for example Mg, are no longer suitable for fabricating bioresorbable bioelectrodes since their dissolution rates are relatively fast. In 2016, Yu et al. utilized highly doped SiNM as the bioresorbable bioelectrodes, which can form conformal contact and stable interface with the complex and curvy surface of neural tissues, for in vivo recordings of electrophysiological signals [25]. Advantages of SiNM-based bioresorbable bioelectrodes mainly lie in their convenience of fabrication, slow dissolution rate, and uniform reduction in thickness during the dissolution. A thin layer of SiO_2_ (thickness of ~100 nm), with windows to expose the defined areas of SiNM electrodes, serves as the dielectric. By attaching a four-channel bioresorbable SiNM bioelectrode array to the cortical surface of the left hemisphere of a rat, the pre-ictal and ictal-like spiking epileptiform activity of the rat could be recorded. Optimizations in thicknesses of SiO_2_ (300 nm) and Si NMs (1000 nm) allow for chronically recording the electrocorticography for more than one month. This operation time is sufficient for clinical usage of monitoring the seizures of epilepsy.

**Figure 5:**
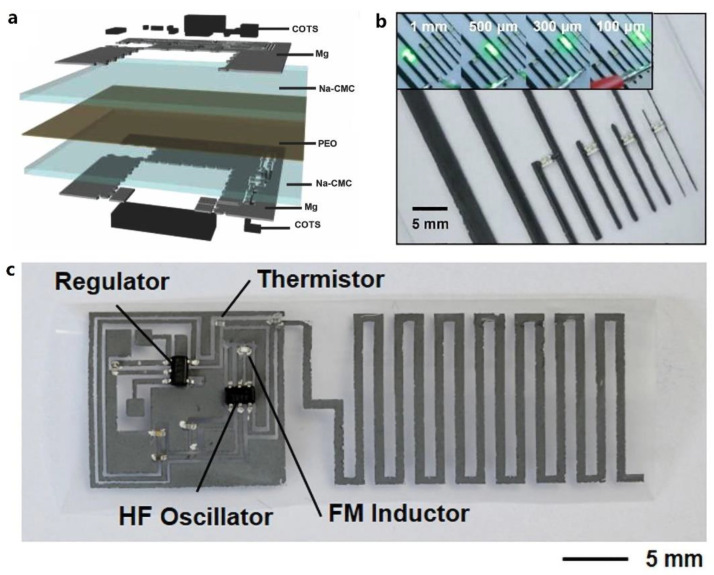
(**a**) Exploded schematic view of a transient printed circuit boards with bioresorbable conductive paste-based interconnections. Reproduced with permission from [97]. (**b**) Optical image of screen-printed conductive lines of Mo/PBTPA paste with different dimensions. Reproduced with permission from [15]. (**c**) Screen-printed bioresorbable radio circuit with Zn/PVA composite paste. Reproduced with permission from [56].

Besides, biodegradable electrodes also play important roles in transient electronics as the interconnection of device array or systems [97], current collectors in energy devices such as solar cells [32], or others. In 2014, Huang et al. developed a transient metal paste, which was a mixture of W and poly (ethylene oxide), as the interconnection bridge for multilayer transient printed circuit boards [97]. As schematically shown in Figure 5a, multilayers of commercial-of-the-shelf components were electrically connected and fixed on Mg contact pads by the developed transient conductive paste. More recently, bioresorbable conductive pastes either with high conductivity or with long-term operation time have been made by other biodegradable materials, such as Mo with poly butanedithiol 1,3,5-triallyl-1,3,5-triazine-2,4,6(1H,3H,5H)-trione pentenoic anhydride (PBTPA) [15], Zn with PVA [56], and W with candelilla wax [75]. One of the most significant advantages for these bioresorbable conductive pastes lies in the ease of fabrication and patterning process. For example, screen-printed technique enables the patterning of paste materials into conductive lines with various dimensions (Figure 5b) [15] or functional bioresorbable radio circuit (Figure 5c) [56]. Moreover, for some specific application scenarios that require flexible or stretchable characteristics of bioresorbable passive devices or systems, mechanical designs such as serpentine and/or noncoplanar interconnections will exhibit great opportunities [24].

## 4. Bioresorbable Capacitors

Capacitor is another basic component of passive electronic devices. The most common structure of a capacitor contains two parallel or faced electrodes separated with a middle dielectric layer. In terms of biomedical applications, bioresorbable capacitors are usually developed as sensors or power supply devices. In this section, capacitor-based bioresorbable sensors and power supply devices (such as supercapacitors) will be reviewed. We will start with an example of capacitor-based bioresorbable sensor that can monitor both pressure and strain, then discuss the bioresorbable supercapacitor as a typical power supply device for bio-implantable electronics.

### 4.1. Pressure/Strain Sensor

**Figure 6:**
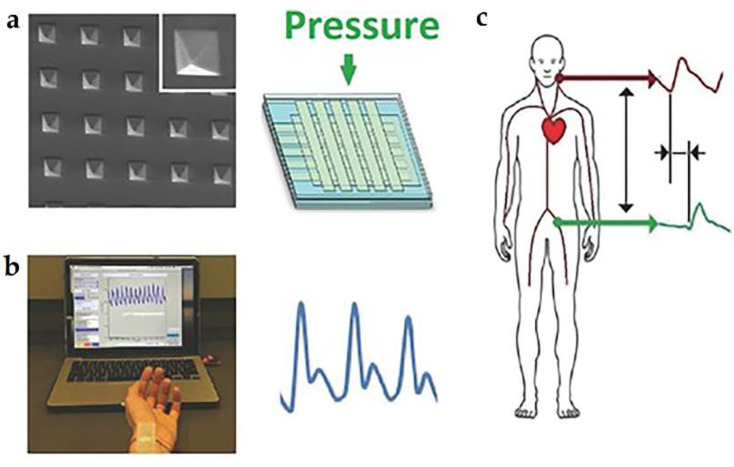
(**a**) SEM images of PGS films with square pyramid structure (**left**) and the device structure of the biodegradable pressure sensor (**right**); (**b**) blood pressure wave of the radial artery measured with the biodegradable pressure sensor; (**c**) collection of blood pulse wave at the femoral and carotid artery for pulse wave velocity measurements. Reproduced with permission from [18].

Parallel-plate capacitors can respond to the external pressure or strain through changes of the distance between two electrodes, the relative dielectric constant, or the effective area of the electrode. As for the biomedical application, highly sensitive pressure sensors are required, especially for the cardiac health, such as the monitor of pulse, arterial waveform, and pulse wave velocity. In 2015, Boutry et al. proposed an array of highly sensitive (~0.76 kPa^−1^) and fully biodegradable pressure sensors for cardiovascular monitoring [18]. As shown in Figure 6a, square pyramid patterned PGS serves as the dielectric between the orthogonal top and bottom electrodes. Facilitated by the minimal viscoelastic performance of PGS, fast response from the pressure sensor is available. Then, the pressure sensor array was made into a single-use patch that can be mounted above the radial artery on the wrist, as displayed in Figure 6b. Furthermore, pulse waves of carotid artery and femoral artery are simultaneously recorded through two pressure sensor arrays. As a result, the pulse wave velocity can be calculated, as schematically illustrated in Figure 6c. This pressure sensor could work stably for thousands of cycles in the air.

In addition to high sensitivity and fast response time, simultaneous measurements of strain and pressure are required for the tissue rehabilitation monitoring. In 2018, Boutry and the collaborators promoted their previous work by using two vertically isolated sensing parts that can simultaneously measure strain and pressure independently [26]. The capacitive bioresorbable pressure sensor consists of a microstructured elastic dielectric layer similar to their previous work, a top electrode encapsulated by PLLA, and a bottom comb electrode supporting on a PLLA substrate. The capacitive bioresorbable strain sensor contains two neighboring bottom comb electrodes that could slide relatively to change the separation distance under the external strain. The proposed bioresorbable sensor exhibits outstanding performances, with a millisecond-range response time and a stable operation for thousands of circles. Encapsulated with POMaC, in vivo long-term operation (over 3.5 weeks) in rat models was also demonstrated.

### 4.2. Supercapacitor

Bioresorbable are promising power supply devices that store and provide energy for bio-implantable electronics with high power density and short time intervals. According to the capacitive behavior, supercapacitors can be divided into two types, i.e., electrical double layer capacitance (EDLC) and pseudocapacitance. The conventional EDLCs can form an electrical double layer at the interface between the electrode and the electrolyte, which is a non-Faradaic process. Pseudocapacitance involves a fast and reversible surface redox reaction, which is a Faradaic process.

**Figure 7:**
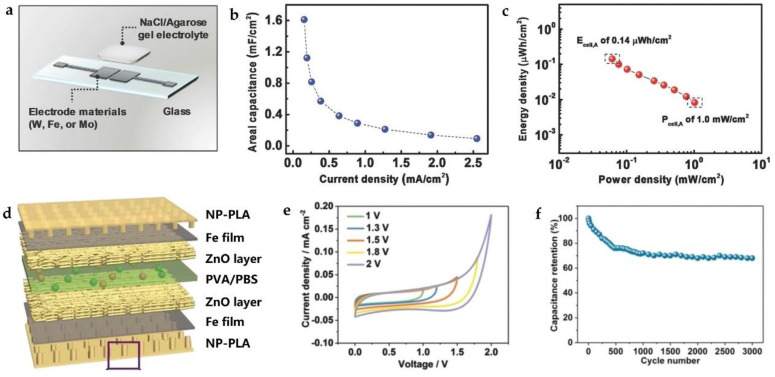
(**a**) Schematic illustration of the biodegradable planer supercapacitor on glass substrate; (**b**) area capacitance calculated according to the charge or discharge curves with different current densities in the range of 0.15 to 2.6 mA/cm^2^; (**c**) Ragone plot of the supercapacitor. Reproduced with permission from [29]. (**d**) Device structure of the multilayered structured biodegradable supercapacitor; (**e**) test of safe operation voltage window; (**f**) fatigue test with the current density of 0.02 mA/cm^2^ at 1 V. Reproduced with permission from [98].

In 2017, Lee et al. reported biodegradable planar microsupercapacitors (MSCs) consisting of bioresorbable metals (W, Fe, and Mo) as the electrodes and NaCl/agarose gel as the electrolytes, as schematically illustrated in Figure 7a [29]. During the charge/discharge cycling, the metal electrode undergoes electrochemical oxidation with the water containing hydrogel electrolyte. By utilizing Mo interdigitated electrodes (thickness ~ 300 nm), the MSC, with a total area of 0.785 cm^2^, offered the highest areal capacitance of 1.6 mF/cm^2^ (at a current density of 0.15 mA/cm^2^), as shown in Figure 7b. And the areal energy density reached 0.14 µW·h/cm^2^ at a power density of 61 µW/cm^2^, while the power density was 1.0 mW/cm^2^ at an energy density of 0.0083 µW·h/cm^2^, as indicated in Figure 7c. The performances of MSC were comparable to the permanent supercapacitors. Moreover, the flexible MSC maintained stable electrochemical performance during convexly and concavely bending processes. Encapsulated with a hydrophobic polyanhydride (PA) film (thickness ~ 150 µm), the MSC can work for 2 weeks in PBS (pH = 7.4, 37 °C).

Two years later, Li et al. proposed a bioabsorbable capacitor that had a longer operation time [98]. The capacitor had a symmetrical multilayered structure that consisted of two nanopillar polylactic acid (NP-PLA) films, two Fe current collectors, two self-assembled ZnO nanoporous layers, and one polyvinyl alcohol/phosphate buffer solution (PVA/PBS) hydrogel as solid-state electrolyte and separator (Figure 7d). PLA with nanopillars provides strong adhesion to the Fe film (~200 nm), and serves as the encapsulation to protect the whole capacitor from the corrosion by biofluids. The ZnO layer (~18 µm) was used for ion storage. As a result, the bioabsorbable capacitor could reach the safe operating voltage up to 1.5 V, as shown in Figure 7e. After 3000 cycles of charge/discharge, about 70% of the capacitance was retained (Figure 7f). The operation lifetime, which is tunable by adopting different encapsulation materials (e.g., PVA and PLA), reaches 30 days in PBS and 50 days in Sprague Dawley rats, respectively. 

Recently, Shang and his collaborators demonstrated a deformable, higher-performance bioresorbable supercapacitors, and a binder-free pseudocapacitance electrode containing amorphous molybdenum oxide (MoO_x_) flakes on Mo foil was introduced [30]. Advantages of this bioresorbable supercapacitor mainly manifest in its mechanical flexibility, tunable dissolution duration, and high areal capacitance (112.5 mF/cm^2^ at 1 mA/cm^2^) and energy density ((15.64 μW·h/cm^2^). Similar to other works, the life span of the bioresorbable supercapacitor after its implantation can vary from a few days to a few weeks through material strategies of the encapsulations.

## 5. Bioresorbable Inductors

Inductor is a two-terminal electronic passive component, offering great potentials for the application in radio frequency (RF) operations, such as data communications and power supplies. For bioresorbable inductors, with a purpose of biomedical applications, the most attractive characteristic lies in their abilities of wirelessly communicating data or supply powers, which eliminate the risks of infection and physical constraints associated with traditional percutaneous wire connection. More often, bioresorbable inductors are integrated with other passive electrical components to form a functional circuit, which will be introduced in the next section. In this section, we will give a typical example of bioresorbable inductors, with an emphasis on their biomedical applications in power supply. 

**Figure 8:**
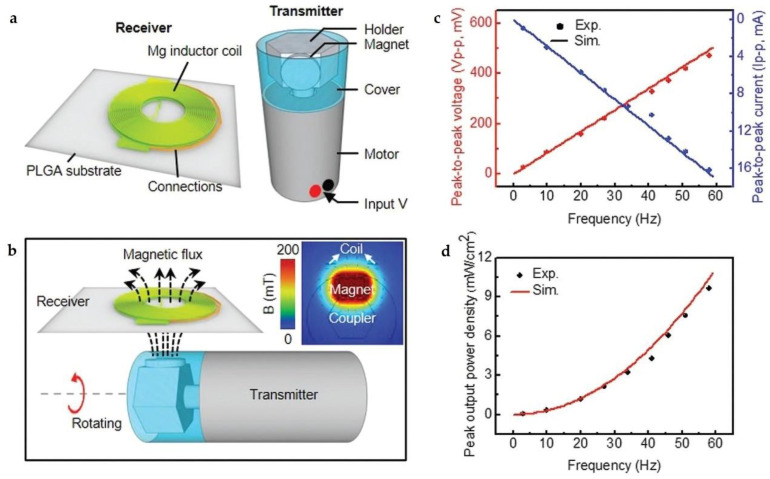
(**a**) Schematic illustration of the bioresorbable antenna and the transmitter; (**b**) illustration of the working principles of the device; (**c**) stimulation and experimental results of open-circuit voltage (red line and red dot, respectively) and short-circuit current (blue line and blue dot, respectively) at different frequencies; (**d**) simulations (red line) and experimental (red dots) results of peak output power densities at different frequencies. Reproduced with permission from [21].

In 2019, Guo et al. demonstrated an inductor-based bioresorbable wireless power supply system, in which a bioresorbable antenna was designed as the receiver to harvest energy from a rotating magnet [21]. As shown in Figure 8a, the bioresorbable antenna consisted of two vertically stacked Mg planar coils (thickness ~ 30 µm) connected in a series and a PLGA interlayer, while the transmitter was formed by a neodymium disc magnet bonded on the holder of a commercial electric motor. The horizontal plane of the antenna was parallel to the surface of the permanent magnet with their centers aligned. According to Faraday’s law, the rotation of the magnet will change the magnetic flux through the antenna, as shown in Figure 8b, thus inducing an electromotive force (i.e., voltage) that strongly depends on the rotational speed. As demonstrated in Figure 8c, the peak-to-peak voltage and the peak-to-peak current increase linearly with the rotational frequency. The output power density is proportional to the square of the frequency, and could reach about 10 mW/cm^2^ at a frequency of 58 Hz, as shown in Figure 8d. Furthermore, a magnetic field concentrator (MFC) made of iron oxide nanoparticles (Fe_2_O_3_, ~50 nm in diameter) and PLGA mixture was introduced to further enhance either the working distance between the bioresorbable antenna and the receiver or the output voltage. The authors demonstrated several unique advantages of the system, most significantly the absence of a need for impedance matching (as is required for conventional electromagnetic harvesters) between the transmitter and receiver and the complete elimination of radiation hazards and electromagnetic absorption by biological tissues.

## 6. Bioresorbable LCR Circuits

The integration of resistors, capacitors, and inductors form an LCR resonant circuit, which can transmit data and receive power wirelessly through the near field inductive coupling. For a bioresorbable LCR circuit, the abilities of sensing bio-signals and supplying powers in a wireless manner represent great potentials for biomedical applications, because of the elimination of infection risks and/or physical constraints caused by transdermal wires. The mechanism of an LCR-based wireless sensor relies on its resonant frequency that depends on the inductance and the capacitance of the circuit. Therefore, bio-signals that change the inductance or the capacitance of a LCR resonant circuit can be quantificationally extracted through the wireless measurement of the resonant frequency. Moreover, similar to the bioresorbable inductor, bioresorbable LCR circuit can act as an electromagnetic harvester that provides electrical powers for bio-implantable electronics, or for electrical stimulations for the treatment of human diseases. In this section, several demonstration examples of bioresorbable LCR circuits, with an emphasis on their biomedical applications, including bio-signals sensing, drug delivery, and neural regeneration, will be described.

### 6.1. Wireless Sensors

In 2013, Luo et al. demonstrated a fully biodegradable wireless pressure sensor, consisting of a variable parallel-plate capacitor connected to an inductor coil [99]. A sensing cavity between the two-parallel plate of the capacitor deforms as the external pressure varies, thus leading to changes in resonant frequency of the circuit. An external reading coil is introduced to wirelessly detect the variations of the resonant frequency. Notably, the authors utilized Zn/Fe bilayers as conductors, because Zn could improve the electrical performance at high frequency and the introduction of Fe could increase its dissolution rate. As a result, the wireless bioresorbable pressure sensor revealed a sensitivity of −39 kHz/kPa in the air (over the range of 0–20 kPa), and a sensitivity of −35 kHz/kPa in saline solution (0.9% NaCl in DI water). Stable operation of the device occurred after the immersion in saline for 20 h, with a sensitivity of −54 ± 4 kHz/kPa, which can last for 86 h.

In vivo demonstrations of the LCR circuit-based bioresorbable wireless pressure sensor are also available. In 2019, Boutry et al. reported a biodegradable cuff-type pressure sensor to monitor the arterial blood flow of patients after complex reconstructive surgeries [27]. The device consisted of a capacitive pressure sensor and an inductor for radio frequency data transmission. The pressure sensor included two narrow fringe-field capacitors, which were fabricated by Mg electrodes (thickness ~ 50 µm) with a pyramid-structured PGS dielectric layer (thickness ~ 40 µm) on it. The inductor consisted of two coils with optimized link designs and a 50-µm-thick PLLA spacer between them. The whole device was packaged with a soft top layer of 10-µm-thick POMaC, which was in contact with the artery and a stiff bottom layer of 10-µm-thick PHB/PHV that exposed to the surroundings, so the pressure sensor was mainly sensitive to the artery expansion caused by pulsed blood flow rather than the respiratory motion. The change in capacitance generated from the artery expansion would result in a shift of the resonant frequency of the LCR circuit. In this case, the resonant frequency could be obtained from the scattering parameter S11, which was wirelessly detected by an external reader coil through inductive coupling. Optimizations in the thickness and the pyramid structure of the dielectric layer, and the separation distance as well as the orientation of two Mg coils enabled a fast response time (millisecond scale), negligible hysteresis, and high reproducibility. The demonstrated bioresorbable wireless pressure sensor could monitor the real-time blood flow both in a custom-made artificial artery and in rat models. Opportunities of bioresorbable wireless pressure sensor for biomedical engineering also include the wireless monitoring of intracranial or intra-abdominal pressures [100], as well as orthopedic applications [101].

**Figure 9:**
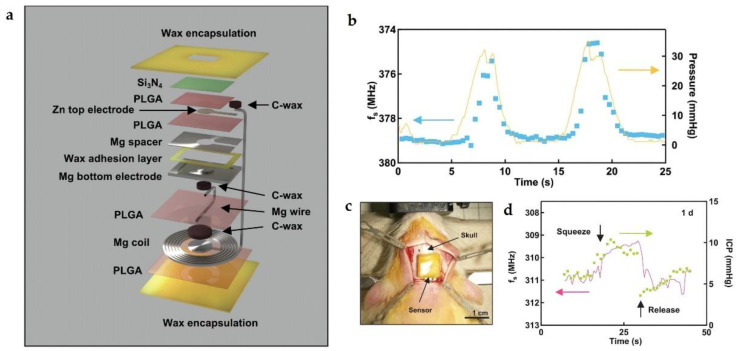
(**a**) Exploded view schematic illustration of the biodegradable wireless pressure; (**b**) in vitro measurements of resonant frequencies with pressure applied on the device; (**c**) image of the sensor implanted in rat before suturing; (**d**) intracranial pressure measured with biodegradable sensor and commercial sensor 1 day after the implantation. Reproduced with permission from [100].

Aiming for biomedical applications, two primary requirements of bioresorbable wireless sensors including tunable operation period, especially for long-term stable operation, and the high performance, especially for ultra-high sensitivity should be satisfied. In 2020, Lu et al. reported a LCR circuit-based bioresorbable wireless sensor, with high performance and long-term operation, for the monitoring of pressure at targeted locations inside animal models [100]. As schematically shown in Figure 9a, the construction of the device mainly includes a Mg inductor coil and an optimized capacitor. As a result, the sensitivity of the pressure sensor reaches as high as 200 kHz/mmHg with a resolution of 1 mmHg, which is sufficient for clinical applications. Both in vitro and in vivo monitoring of the intracranial pressure by utilizing the proposed biodegradable pressure sensor are comparable to those obtained from a clinical standard device, as shown in Figure 9b,d. Moreover, an inorganic layer of Si_3_N_4_ (2 µm) was deposited on the top electrode, with the edges sealed with nature wax (about 500 µm), serving as an excellent flexible biofluid barrier. Therefore, the device offered stable in vivo operations for 4 days, and leaving only the Si_3_N_4_ membrane and the wax encapsulation after 44 days.

Temperature variations inducing changes in the dielectric constant of capacitor form the basis for the LCR-based bioresorbable wireless temperature sensor. In 2020, Lu et al. proposed a bioresorbable wireless temperature sensor with potentials for tracking the temperature variation within human body [17]. The wireless sensor contains a separated Mg inductor coil and a temperature sensitive capacitor. PLA was used as spacers of the capacitor, and its dielectric constant was sensitive to temperature, so the resonant frequency of the LCR circuit was strongly depended on the temperature. The encapsulation was completed by PLGA (10 μm thick) and nature wax, allowing reliable operation for 4 days in rats. Notably, the inductor with a relatively large size enables a long readout distance (about 1.2 cm), while the capacitor with a small size is favored for the measurement of local temperature.

### 6.2. Drug Delivery

**Figure 10:**
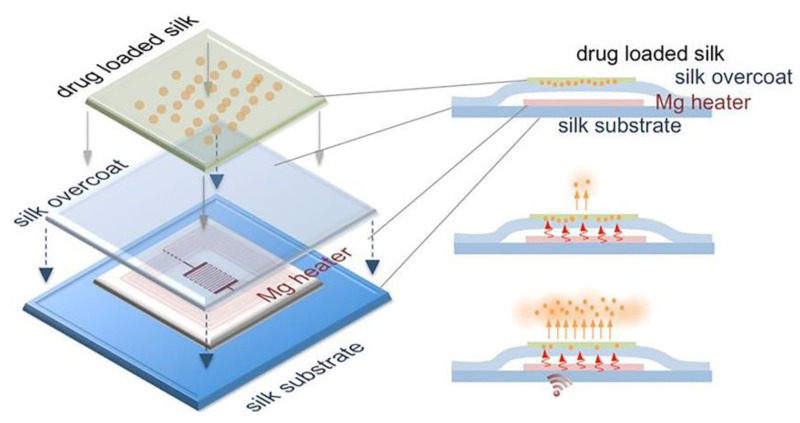
Schematic illustration of the biodegradable wireless drug delivery. The release of antibiotics (orange spot) loaded on silk film (green substrate) would be enhanced by the remote-controlled Mg heater. Reproduced with permission from [8].

Localized drug delivery offers advantages over systemic administration in some pharmacological therapies like hormone imbalances adjustment, brain tumor treatment, and diabetic care. In 2014, Tao et al. proposed a remotely controlled drug delivery system constructed with a LCR circuit, as shown in Figure 10 [8]. A bioresorbable Mg inductor coil, a bioresorbable Mg resistor, a silk substrate, and a drug loaded silk membrane formed the wireless drug delivery system. The ability to wirelessly harvesting the electromagnetic energy by the inductor coil causes the rise in temperature of the resistor, thus enabling the accelerated release of the loaded drugs (herein, antibiotics) from silk. The drug releasing rate and dose could be also affected by the crystallinity of the silk, which was controlled by the boiling time of raw silk fibers and the water annealing time of cast silk films. Finally, the wireless system could be fully degraded and resorbed after the accomplishment of the drug delivering process.

Programmable drug delivery is also demonstrated by a bioresorbable wireless system with multiple, independently controlled reservoirs which could store single or multiple classes of drugs. The system consists of an array of inductor coils, each of which connects to a serpentine resistor (for Joule heating), and thermally actuated lipid membranes embedded with drugs. And the lipid membrane was designed to have long-period ability of retaining drugs with minimal leakage at body temperature, as well as the ability of rapidly releasing drugs at heating temperature (41–43 °C). Optimizations in the configurations of the coil array enable independent and programmable release of drugs, which is promising for clinical applications. In other cases, such as brain tumor treatment, orientational drug release is needed. To address this issue, Lee et al. reported a flexible, temperature-triggered drug delivery patch with sticky/hydrophobic bifaces, which offered wirelessly controlled, spatially focused, and temporally extended delivery of antitumor agents up to deeply located brain tumors [9].

The wireless drug delivers mentioned above all relied on hypothermic platforms, so they might be affected by the temperature fluctuation inside the human body. In addition, unwanted leakage of drugs at the off state remains a problem due to the biodegradability of the reservoir materials. In 2020, Koo et al. proposed a wireless drug delivery system using the electrochemical method to cut down the off-state leakage [10]. The system consists of a wireless power harvester with Mg coils as the receiver, a metal gate valve, and a polymer reservoir. Upon the activation of an RF transmitter (external coils), the wireless power harvester generates an overpotential that results in an accelerated electrochemical etch of the metal gate valve. As a result, triggerable release of drugs through the opened gate is realized. Moreover, optimizations in an array of wireless power harvesters, each of which has different resonant frequencies, can lead to a wirelessly programmable release of insulin for the regulation of blood glucose [7].

### 6.3. Neural Regeneration

Electrical stimulation of the proximal portion of an injured nerve is helpful for accelerating the functional recovery. It provides a way of nonpharmacological, bioelectric therapy. In 2018, Koo et al. demonstrated a wireless, bioresorbable electrical platform, constructed with a bilayer of Mg inductors with PLGA membrane as the interlayer, a SiNM-based RF diode, a parallel plate capacitor with Mg as the electrodes, and an electrical interface exposed to the targeted peripheral nerve [6]. The RF power harvested by the coils was sent to the encircled region of the nerve, turning out that the electrical stimulation of peripheral nerve accelerates the neural regeneration. This remotely controllable, bioresorbable platform, encapsulated with PLGA film, could last for 6 days during the in vivo experiment. In some cases, however, this operation time may be insufficient for nerve to exceed the interruption caused by many traumatic injuries. More recently, the same group developed a long-lived, stretchable, and wireless bioresorbable electrical stimulator [102]. A critical biotechnology for constructing this system manifests in the special synthesis of bioresorbable dynamic covalent polyurethane (b-DCPU) that offers mechanical stretchability and minimal swelling in biofluids. Therefore, robust operation in moving tissues, with the limitation on lifetimes, was achieved by utilizing d-DCPU as the substrate and biofluid barriers. As a result, in vivo experiment demonstrated the long-term electrical stimulation of sciatic nerves in rat models over 30 days, which exceeds the timeframes for the recovery of traumatic nerve injuries.

## 7. Summary and Outlook

In this review, we have summarized the recent progress on materials, device architectures, and integration strategies of bioresorbable passive electronics, with emphases on their applications in biomedical engineering. During the past decade, great efforts have been devoted to the invention and promotion of various types of bioresorbable passive electrical devices and/or systems with different functionalities, probably because of their simple device structures that allow for convenient fabrications, and the straightforward working principles that enable precise data/signal analyses especially important to the diagnosis and treatment in biomedical applications. Compared with some typical implantable commercial sensors, the currently demonstrated bioresorbable passive sensors, such as intracranial pressure sensor [5], intracranial temperature sensor [17], and pH sensor [24], exhibit comparable performances, including the measurement range or even the precision. Moreover, the secondary surgery for the removal of implanted electronics can be eliminated for bioresorbable passive sensors. In addition, bioresorbable passive devices are normally fabricated onto bioresorbable substrate, which is ultra-thin and naturally flexible, thus bypassing the large mechanical mismatch between devices and bio-tissue and reducing the passible damage and discomfort to bio-tissue. However, the limited stable operation time, which is induced by the fast dissolution rate of materials and device, is still a significant challenge for the practical applications of bioresorbable passive sensors. Possible approaches to address this issue includes materials and encapsulation strategies to reduce the dissolution rate or prevent the penetration of bioliquids or ions.

Future opportunities of this area lie in many aspects, including the following: (i) expanding the sensible range for other bio-signals (including biophysical and biochemical signals, as well as biopotentials), since most of present bioresorbable sensors by passive devices are developed for the sense of common bio-signals, such as pressure, temperature, and biopotentials, though critical for human health status; (ii) realizing the simultaneous sense of multiple bio-signals with precise decoupling from a single device; (iii) realizing on-demand transience of bioresorbable devices through triggerable stimuli; (iv) developing new materials or designing novel structures of encapsulations to control the stable operation time (ranging from several days, weeks, months, to years) after the implantation; (v) fabricating suitable bioresorbable wireless energy harvesting or storage devices to supply electrical powers for bioelectronic implants.

For the LCR circuit formed by the integration of different bioresorbable passive electronic devices, future opportunities lie in many aspects of science, engineering, and practical applications, including the following: (i) simultaneously sensing multiple bio-signals with precise decoupling from a single unit; (ii) optimizing circuit designs to attain robust sensing performance without any drift resulted from the variations in readout distance, clinical environments, or misalignment between the readout coil and the LCR sensor; (iii) increasing the readable distance is vital for their practical biomedical applications; (iv) reducing the overall size (millimeter scale) of a LCR circuit that suitable for minimally invasive implantation. These fascinating functionalities and unique advantages of bioresorbable passive electronic devices and/or systems, as well as great opportunities and challenges for biomedical applications, will promote researchers from both academic organizations and industrial companies to put unremitting efforts, which in return stimulates the development of material science, sensor metrics, bioinspired electronics, and others.

## Figures and Tables

**Table 1:** Key materials for bioresorbable passive devices.

Materials		Name	Application	Dissolution Rate	Dissolution Condition	Reference
Inorganic Materials	semiconductors	Si ^1^	active material	4.5 nm/day	in PBS at pH 7.4 and 37 °C	[23]
		Ge		3.1 nm/day	in buffer solutions at pH 7.4 and 37 °C	[32]
		SiGe		0.1 nm/day	in buffer solutions at pH 7.4 and 37 °C	[32]
		ZnO		100 nm/day	in PBS at pH 7.4 and 37 °C	[37]
	conductors	Mg	electrode, antenna, interconnection materials	0.05–0.5 μm/h	in Hanks’s balanced saline solution (HBSS) at 37 °C	[43]
		Zn		5 × 10^−3^ μm/h	in vivo	[43]
		W		0.02–0.06 μm/h	in HBSS at room temperature (RT)	[43]
		Fe		0.02 μm/h	in HBSS at 37 °C	[43]
		Mo		2 nm/day	in PBS at pH 7.4 and 37 °C	[4]
	dielectrics	SiO_2_ ^2^	interlayer dielectrics and biofluid barrier	1.4 nm/day	in PBS at pH 7.4 and RT	[4]
		Si_3_N_4_		0.16 nm/day	in buffer solutions at pH 7.4 and 37 °C	[44]
Organic Materials	semiconductors	FF:PF	active material	-	-	[41]
		RFLT	active material	-	-	[42]
	conductors	CS-GA-AT ^3^	electrode	12.9% weight loss/48 h	in buffer solutions at pH 7.4 and RT	[44]
	dielectrics	PGS (50/50)	substrate	8.6%/day	in PBS at pH 7.4 and 37 °C	[2]
		PLGA (50/50)	substrate	2%/day	in PBS at pH 7.4 and 37 °C	[45]

^1^ The degradation rate varies from 0.3 nm/day to 100 nm/day, depending on morphology, the type and concentration of dopants, temperature, pH, and concentrations of albumin, Si(OH)_4_ and ionic in dissolution environment [23,31,32,33,34,35,36]. ^2^ The degradation rate varies from 4.7 × 10^−4^ μm/h to 282 nm/day, depending on pH, ion concentrations, temperatures, and deposition methods [44]. ^3^ Chitosan cross-linked to the aniline tetramer segment by glutaraldehyde.

## Data Availability

Data sharing is not applicable to this article.
